# Supplementary material for: Seawater Reverse Osmosis Performance Decline Caused by Short-Term Elevated Feed Water Temperature
Source: Membranes (Basel). 2022 Aug 18;12(8):792. doi: 10.3390/membranes12080792 (PMC9416791; doi:10.3390/membranes12080792)
Supplement: Supplementary file 1 [file membranes-12-00792-s001.zip › membranes-1855313-supplementary.pdf]

membranes

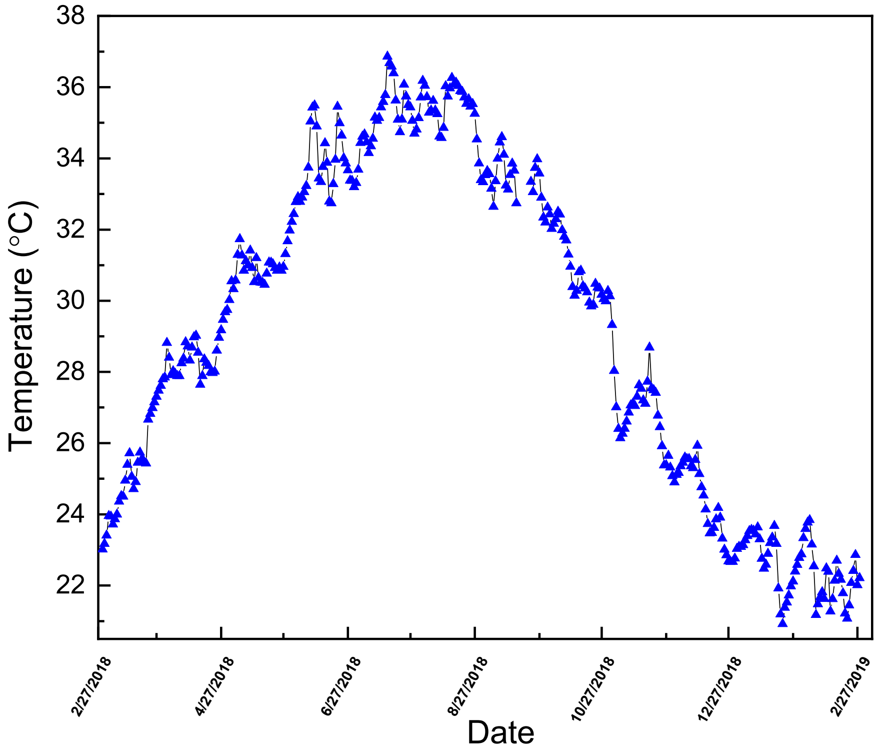

Figure S1: Average daily temperature for the year 2018–2019 for seawater at a full-scale desalination plant in the Arabian Gulf.

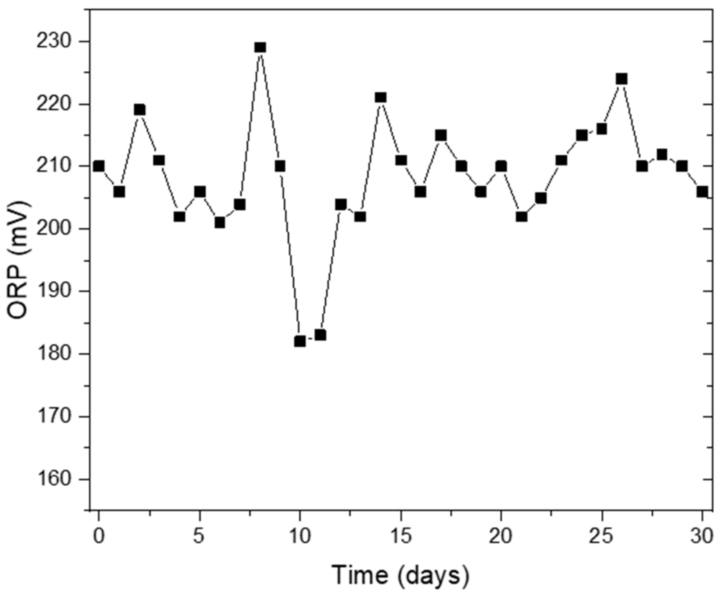

Figure S2: The oxidation reduction potential (ORP) profile of the seawater feed used for the pilot test. ORP was constant, indicating no membrane damage due to chlorination.

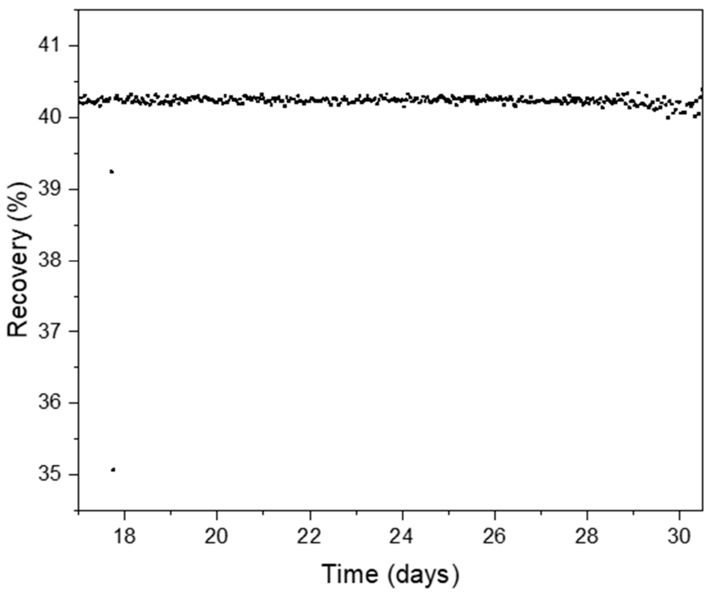

Figure S3: Pilot operation at a constant recovery of 40%.

Section S1: Membrane Performance Normalization

Normalization is a technique that allows for comparing the operation at a specific set of conditions to a reference set of conditions [1,2]. This enables the actual plant performance to be understood and helps determine whether changes in flow or salt rejection are

due to fouling, membrane damage, or are just due to different operating conditions. In other words, data normalization distinguishes a fouling state from a non-fouling/normal state.

Normalized permeate flow (NPF), normalized salt passage (NSP), and normalized differential pressure (NDP) are calculated as follows [3]:

Permeate and feed–brine osmotic pressures used in NDP are calculated as follows:

$$\pi_P(bar) = \frac{1.01327 \times 0.082054 \times (T + 273.15) \times 2 \times 1000 \times 2 \times C_P}{1000 \times 58.44 \times 2 \times 1000} \times \left(10^{\frac{-0.5\times\sqrt{\frac{1\times C_P\times 2\times 1000\times 1^2\times 4}{2\times 2\times 1000\times 1000\times 58.44\times 2}}}{1+\sqrt{\frac{1\times C_P\times 2\times 1000\times 1^2\times 4}{2\times 2\times 1000\times 1000\times 58.44\times 2}}}} \right)^{0.14}$$

Similarly,

$$\pi_{FC}(bar) = \frac{1.01327 \times 0.082054 \times (T + 273.15) \times 2 \times 1000 \times 2 \times C_{FC}}{1000 \times 58.44 \times 2 \times 1000} \times \left(10^{\frac{-0.5\times\sqrt{\frac{1\times C_{FC}\times 2\times 1000\times 1^2\times 4}{2\times 2\times 1000\times 1000\times 58.44\times 2}}}{1+\sqrt{\frac{1\times C_{FC}\times 2\times 1000\times 1^2\times 4}{2\times 2\times 1000\times 1000\times 58.44\times 2}}}} \right)^{0.14}$$

where T is the temperature in ^{0}C, C_P is the permeate *TDS* (mg/L) calculated using the *EC-TDS* conversion (described later), and C_{FC} is the feed–brine average concentration calculated as follows:

$$C_{FC}\ (mg/L) = C_F(mg/L) \times \frac{1 - (1 - Y)^{(1-SR)}}{(1 - SR) \times Y}$$

where Y and SR are fractions that represent nominal recovery (at any time t) and nominal salt rejection at time = t calculated as follows:

$$Y = \frac{Q_P(m^3/h)}{Q_F(m^3/h)}$$

$$SR = 1 - \frac{C_P\ (mg/L)}{C_F(mg/L)}$$

where Q_P and Q_F are the permeate and feed flowrates, respectively. Similarly, C_P and C_F represent the permeate *TDS* (mg/L) and feed *TDS* (mg/L), respectively. C_P and C_F are calculated in mg/L using the same correlation and coefficients as follows:

- If *EC* (µS/cm) is greater than 7630 (*EC* > 7630), then

$$C\ (mg/L) = uSa \times e^{\frac{\left(uSb - Ln\left(e^{(0.0017\times(T-25))}\times EC\right)\right)^2}{uSc}}$$

- If *EC* (µS/cm) is less than 7630 (*EC* < 7630), then

$$C\ (mg/L) = uS2a \times e^{\frac{\left(uS2b - Ln\left(e^{(0.0017\times(T-25))}\times EC\right)\right)^2}{uS2c}}$$

where C is the concentration in mg/L (feed or permeate), and *EC* is the electrical conductivity in µS/cm (feed or permeate) inputted daily. The table below shows the coefficients used for the *EC* to *TDS* conversion,

Table S1: *EC-TDS* conversion coefficients

**uSa**	0.000000000080090966
**uSb**	-50.645805186
**uSc**	112.483950289
**uS2a**	7.7013840097E-20
**uS2b**	-90.475562243
**uS2c**	188.88442227

Temperature Correction Factor (TCF) used for NPF is calculated as follows:

- If Temperature is less than or equal to 25 ^{0}C ($T \leq 25$), then

$$TCF_{Flow} = \mu \times e^{TempA1 \times (T-25)} \times \left(\frac{T_K}{298}\right)^{\frac{TempA3}{1-Y}}$$

- If Temperature is greater than 25 ^{0}C ($T > 25$), then

$$TCF_{Flow} = \mu \times e^{TempA2 \times (T-25)} \times \left(\frac{T_K}{298}\right)^{\frac{TempA4}{1-Y}}$$

where Y is the nominal recovery described above, and μ is water viscosity (factor) calculated as follows:

$$\mu = \frac{e^{\frac{1965}{298.15}}}{e^{\frac{1965}{T_K}}}$$

where T_K is the temperature in Kelvin as follows:

$$T_K = T + 273.15$$

TempA1, TempA2, TempA3, TempA4 are membrane model related parameters available from the membrane OEM (Table S3).

NSP is calculated as follows:

$$SP_n = SP_t \times \frac{Q_{P,t} \times TCF_{Salt,0} \times C_{FC,0} \times C_{F,t}}{Q_{P,0} \times TCF_{Salt,t} \times C_{FC,t} \times C_{F,0}}$$

The second way to calculate normalized salt passage is to replace C_{FC} by CF_{lm} as follows:

$$SP_n = SP_t \times \frac{Q_{P,t} \times TCF_{Salt,0} \times CF_{lm,0} \times C_{F,t}}{Q_{P,0} \times TCF_{Salt,t} \times CF_{lm,t} \times C_{F,0}}$$

where Q_P is permeate flowrate ($t = t/t = 0$), C_{FC} and C_F are calculated as described above, and SP_t is the actual Salt Passage calculated as follows:

$$SP_t = \frac{C_{P,t}(mg/L)}{CF_{lm,t}(mg/L)}$$

where C_P is the calculated permeate *TDS* (mg/L) as described above, and CF_{lm} is the feed–brine average log mean calculated at any time (t=t/t=0) as follows:

$$CF_{lm} = \frac{C_C(mg/L) - C_F(mg/L)}{\ln\left(\frac{C_C(mg/L)}{C_F(mg/L)}\right)}$$

where C_F is the calculated feed *TDS* described above, and C_C is the concentrate *TDS* calculated using mass balance as follows:

$$C_C = \frac{C_F Q_F - C_P Q_P}{Q_C}$$

where C represents concentrations at any time (t=t/t=0), and Q describes flowrate of feed, brine, and permeate. TCF_{Salt} is the temperature correction factor used for both normalized salt passage equations (different from TCF_{Flow}) and is calculated as expressed below:

- If Temperature is less than or equal to 25 ^{0}C ($T \leq 25$), then

$$TCF_{Salt} = \frac{\mu_{25}}{\mu_T} \times e^{TempB1 \times (T-25)} \times \left(\frac{T_K}{298}\right)^{\frac{TempB3}{1-Y}}$$

- If Temperature is greater than 25 ^{0}C ($T > 25$), then

$$TCF_{Salt} = \frac{\mu_{25}}{\mu_T} \times e^{TempB2 \times (T-25)} \times \left(\frac{T_K}{298}\right)^{\frac{TempB4}{1-Y}}$$

where T_K and Y are temperature in Kelvin and nominal recovery described above. *TempB1, TempB2, TempB3, TempB4* are membrane model related parameters (Table S3). μ_{25} and μ_T are feed–brine average viscosities at temperature 25 ^{0}C and at temperature T (daily input), respectively. The feed–brine average viscosity at 25 ^{0}C can be calculated as follows:

$$\mu_{25} = 1.234 \times 10^{-6} \times e^{\frac{0.00212 \times CF_{lm} \times \rho_{25}}{1000 \times 1000} + \frac{1965}{273.15+25}}$$

where CF_{lm} is the feed–brine average log mean described above, and ρ_{25} (kg/m^3) is the feed–brine average density at 25 ^{0}C calculated as follows:

$$\rho_{25} = \frac{1000 \times \left(1 + \frac{0.00714 \times CF_{lm}}{10000}\right)}{\frac{3.1975 + \left(-0.315154 \times \left((647.27 - (25+273.15))^{\frac{1}{3}}\right)\right) + \left(-0.001203374 \times (647.27 - (25+273.15))\right) + \left(0.000000000000748908 \times (647.27 - (25+273.15))^4\right)}{1 + \left(0.1342489 \times (647.27 - (25+273.15))^{\frac{1}{3}}\right) + \left(-0.003946263 \times (647.27 - (25+273.15))\right)}}$$

Similarly, feed–brine average viscosity at temperature T is calculated as follows:

$$\mu_T = 1.234 \times 10^{-6} \times e^{\frac{0.00212 \times CF_{lm} \times \rho_T}{1000 \times 1000} + \frac{1965}{273.15+T}}$$

where ρ_T (kg/m^3) is calculated as follows:

$$\rho_{25} = \frac{1000 \times \left(1 + \frac{0.00714 \times CF_{lm}}{10000}\right)}{\frac{3.1975 + \left(-0.315154 \times \left((647.27 - (T + 273.15))^{\frac{1}{3}}\right)\right) + \left(-0.001203374 \times (647.27 - (T + 273.15))\right) + \left(0.000000000000748908 \times (647.27 - (T + 273.15))^{4}\right)}{1 + \left(0.1342489 \times (647.27 - (T + 273.15))^{\frac{1}{3}}\right) + \left(-0.003946263 \times (647.27 - (T + 273.15))\right)}}$$

where CF_{lm} is the feed–brine average log mean described above, and T is the daily measured temperature.

NDP is calculated as follows:

$$DP_n(bar) = DP_t(bar) \times \left(\frac{Q_{FC,0}}{Q_{FC,t}}\right)^{DeltaP3} \times \left(\frac{\mu_{T,0}}{\mu_{T,t}}\right)^{DeltaP6}$$

where DP is the differential pressure calculated in the previous section ($P_F - P_C$), μ_T is the feed brine average viscosity shown above, and Q_{FC} is the feed–brine average flow calculated at any time (t=t/t=0) as follows:

$$Q_{FC}(m^3/h) = \frac{Q_F(m^3/h) + Q_C(m^3/h)}{2}$$

where Q_F and Q_C are feed and concentrate flowrate describes previously. *DeltaP3* and *DeltaP6* are membrane model related parameters that are provided my membrane OEM (Table S3).

Specific Flux (Water Permeability) can be calculated in L/(m^2.h.bar) at any point in data normalization as follows:

$$Specific\ Flux\left(\frac{LMH}{bar}\right) = \frac{Q_{P,t}\left(\frac{m^3}{h}\right)}{Area_{Total}(m^2)}$$

where total area is calculated as follows:

$$Area_{Total}(m^2) = Element\ Area\ (m^2) \times No.of\ elements\ per\ stage$$

Table S2: Correction Factors used for normalization

**0.03851**	0.03714	-5.6466	-6.6743	0.00712	0.01921	3.88072	2.35894	1.443389	0.67238

Section S2: Analysis of membrane fouling

Table S1 below presents the elemental composition of the foulant collected from the feed spacer and the membrane surface. The primary cause of fouling was due to the deposition of rust from the pilot plant piping, as indicated by the high presence of Iron, Nickel, and Chromium. As the pilot was operated at a nominal recovery rate of 40%, there was minimal scaling from salts of Calcium, as indicated by the data below. The membrane autopsy also indicated the absence of any ATP, hence ruling out the possibility of any biofouling. Concerted computational investigations are needed to explain such fouling behaviors. However, this is beyond the scope of this work. This has been explored by other researchers [4-7].

Table S3: Elemental composition of the sticky brown deposit on the feed spacer and membrane surface.

Element	value (µg/l)	Element	value (µg/l)
**Be**	<0.001	**As**	<0.001
**B**	<0.001	**Rb**	1.96
**Na**	4264	**Zr**	3.23
**Mg**	791	**Mo**	14.1
**Al**	<0.001	**Pd**	<0.001
**K**	159	**Ag**	1.45
**Ca**	255	**Cd**	<0.001
**Ti**	<0.001	**In**	0.098
**V**	18.7	**Sn**	<0.001
**Cr**	4479	**Sb**	<0.001
**Mn**	62.7	**Ba**	4.40
**Fe**	8600	**W**	<0.001
**Co**	10.9	**Pt**	1.82
**Ni**	617	**Tl**	<0.001
**Cu**	13.9	**Pb**	19.63
**Zn**	13.8	**U**	3.34

References

1. Safar, M.; Jafar, M.; Abdel-Jawad, M.; Bou-Hamad, S. Standardization of RO membrane performance. *Desalination* **1998**, *118*, 13-21.
2. Zhao, Y.; Taylor, J.S. Assessment of ASTM D 4516 for evaluation of reverse osmosis membrane performance. *Desalination* **2005**, *180*, 231-244.
3. TORAY INDUSTRIES, I. TorayTrak. Available online: https://www.water.toray/knowledge/tool/trak/ (accessed on July 7, 2022).
4. Ruiz-García, A.; Nuez, I. Long-term performance decline in a brackish water reverse osmosis desalination plant. Predictive model for the water permeability coefficient. *Desalination* **2016**, *397*, 101-107, doi:https://doi.org/10.1016/j.desal.2016.06.027.
5. Farhat, N.M.; Vrouwenvelder, J.S.; Van Loosdrecht, M.C.M.; Bucs, S.S.; Staal, M. Effect of water temperature on biofouling development in reverse osmosis membrane systems. *Water Res.* **2016**, *103*, 149-159, doi:https://doi.org/10.1016/j.watres.2016.07.015.
6. Jawor, A.; Hoek, E.M.V. Effects of feed water temperature on inorganic fouling of brackish water RO membranes. *Desalination* **2009**, *235*, 44-57, doi:https://doi.org/10.1016/j.desal.2008.07.004.
7. Jin, X.; Jawor, A.; Kim, S.; Hoek, E.M.V. Effects of feed water temperature on separation performance and organic fouling of brackish water RO membranes. *Desalination* **2009**, *239*, 346-359, doi:https://doi.org/10.1016/j.desal.2008.03.026.
